# An experimental test for indirect benefits in *Drosophila melanogaster*

**DOI:** 10.1186/1471-2148-7-36

**Published:** 2007-03-09

**Authors:** Howard D Rundle, Anders Ödeen, Arne Ø Mooers

**Affiliations:** 1 Department of Biological Sciences, Simon Fraser University, Burnaby, BC, V5A 1S6, Canada; 2 Department of Biology & Centre for Advanced Research in Environmental Genomics, University of Ottawa, Ottawa, ON, K1N 6N5, Canada; 3 Department of Animal Ecology, Uppsala University, Norbyvägen 18D, S-752 36 Uppsala, Sweden

## Abstract

**Background:**

Despite much empirical attention, tests for indirect benefits of mate choice have rarely considered the major components of sexual and nonsexual offspring fitness relevant to a population. Here we use a novel experimental design to test for the existence of any indirect benefits in a laboratory adapted population of *D. melanogaster*. Our experiment compared the fitness (mating success, longevity, and productivity) of individuals possessing genomes that derived two generations previously from males that were either entirely successful (studs) or wholly unsuccessful (duds) at achieving mates in three subsequent rounds of mating trials.

**Results:**

Males from the stud treatment were 30% more successful on average at securing mates than males from the dud treatment. In contrast, we found no difference between treatments in measures of productivity or of longevity when measured in a mixed-sex environment. In the absence of females, however, males in the stud treatment outlived males in the dud treatment.

**Conclusion:**

Our results suggest that mating with successful males in this population provides an indirect benefit to females and that, at least in this environment, the benefit arises primarily through the production of more attractive male offspring. However, it is unclear whether this represents solely a traditional sexy sons benefit or whether there is an additional good genes component (with male offspring simply allocating their surplus condition to traits that enhance their mating success). The lack of any detectable differences in female fitness between the two treatments suggests the former, although the longevity advantage of males in the stud treatment when females were absent is consistent with the latter. Determining the effect of this indirect benefit on the evolution of female mate preferences (or resistance) will require comparable data on the direct costs of mating with various males, and an understanding of how these costs and benefits integrate across generations and vary among environments.

## Background

A comprehensive understanding of the evolution of mate choice requires thorough knowledge of the costs and benefits, both direct and indirect, that arise from it [1-3]. The relative magnitudes of these effects is a subject of much current empirical interest. For example, considerable evidence has accumulated from a number of species demonstrating that females can suffer direct harm from their interactions with males [4]. This is especially true in *Drosophila melanogaster*, for which extensive data have demonstrated that males harm females during courtship and mating [4-11]. This male-induced harm causes selection in females to resist it and, if the resulting female counter-adaptations reduce male mating and/or fertilization success, a process of antagonistic co-evolution between the sexes, driven by interlocus sexual conflict, may result [4,12-15]. Such a process appears to be ongoing in at least one well studied *D. melanogaster *laboratory population [9,16-18].

In contrast to such direct costs, whether females also gain a net indirect benefit from their choice of mates is poorly understood. Known as good genes mate choice, theory predicts that, because of a positive genetic correlation between a male's attractiveness and his condition, females mating with attractive males gain an indirect benefit by passing the male's superior genes on to their offspring [1,19,20]. Good genes mate choice requires that male display traits are honest indicators of a male's overall genetic quality or condition [20-25]. This is suggested to be true whenever sexual display traits are costly to produce and their exaggeration increases male mating success because, once genetic variation in the display trait is exhausted, variation in numerous other loci that affect overall condition will be recruited via a process known as 'genic capture' [26]. Because higher condition individuals are better able to pay the cost of trait exaggeration, display traits become honest indicators of male condition. The extent to which display traits are condition dependent, however, is unresolved [27].

Much attention has been directed at good genes indirect benefits because their magnitude is key to determining the net fitness effects of mate choice, especially in systems in which direct benefits are weak or lacking and direct costs exist (e.g., *D. melanogaster*). Contrasting theoretical analyses have suggested that good genes indirect benefits are capable of overcoming direct costs of mate choice [28], or that direct selection on mate choice (arising from its costs or benefits to females themselves), will commonly overwhelm any indirect benefits [29-31]. Empirical data on the magnitudes of these costs and benefits will therefore be crucial to evaluating these different perspectives [3,32].

Results of empirical tests of the magnitude and nature of indirect benefits are mixed. While there are many data demonstrating that mating with attractive males can benefit specific components of offspring fitness [33-42], in some cases other components suffer [33,37,38]. In addition, in the majority of studies key components of sexual and nonsexual fitness were not considered, meaning the net indirect benefit of mating with attractive males could not be determined. A related approach, in which the opportunity for mate choice is manipulated and then the consequences for offspring fitness are measured (either in the next generation or after a number of generations of experimental evolution), has also produced inconsistent results. Benefits to offspring viability, for example, have been found in some studies but not in others [43,44]. Finally, quantitative genetic studies estimating the genetic correlations necessary for indirect selection to occur (i.e. genetic correlations between female preferences and male display traits, and male display traits and offspring fitness) have also provided mixed results [33,45,46], although data of this type are sparse.

The focus of past studies on specific components of offspring fitness occurred in part because it was thought to be a means of distinguishing between two types of indirect benefit: good genes and an alternative 'sexy sons' process in which the indirect benefits of mate choice for a female arise solely from her production of more attractive sons [29,47]. The idea was that improved offspring viability (survival) was indicative of good genes, whereas increased attractiveness (mating success) of male offspring was indicative of a sexy sons process. However, as recent theory has stressed, this is wrong for two reasons [2,29,48,49]. First, under a good genes process, individuals may be able to allocate condition dynamically to different fitness components at the cost of others; an increase in mating success could therefore give the mistaken appearance of a sexy sons process [2,29,49,50]. Second, good genes and sexy sons processes are likely not mutually exclusive [4]. This is because some form of sexy sons indirect benefit is inherent whenever mate choice occurs (assortative mating generates a genetic correlation between display traits and preferences, thereby causing indirect selection for the preference [1,29,51]); there may also be few traits unaffected in some way by overall condition [4,29], although there are little data with which to evaluate the extent of such a relationship [27].

How fitness should be measured is a controversial topic [52-54]. As stressed above, however, the existence of indirect benefits of mate choice cannot be evaluated using data that examine a restricted subset of fitness components: long term success in future generations is the ultimate currency [2]. Although lifetime fitness may be experimentally intractable, effort must clearly be made to simultaneously consider the major components of sexual (i.e. mating success) and non-sexual (i.e. survival/viability and fecundity) fitness relevant to a population. This has been attempted in just two cases. In the first, results from a series of experiments using the well-studied LH_M _laboratory population of *D. melanogaster *have suggested that both sexy-sons and good genes indirect benefits are lacking [17,55,56]. In contrast, another experiment using house crickets concluded that significant indirect benefits existed, primarily in the form of sexy sons, and that these may even be sufficient to offset estimated direct costs [54].

Here we use a novel experimental design to test for the existence of any indirect benefits in a laboratory-adapted population of *D. melanogaster *(Commercial Avenue strain). Our approach involves measuring major components of sexual and non-sexual fitness relevant to their laboratory environment on two sets of individuals: those with genomes that trace their ancestry to males that were highly successful at obtaining mates (stud males), and those with genomes that trace their ancestry to males that were entirely unsuccessful at obtaining mates (dud males). Three successive rounds of mating trials are used to sort males into these two groups (stud males being those that were successful at obtaining mates in all three rounds and dud males being those that were thrice unsuccessful). These males are then mated individually to random females from the same population; their offspring are again mated as random pairs within the stud and dud treatments (in a systematic manner to avoid inbreeding) to form grandchildren possessing genomes that trace their ancestry ultimately to either stud (successful) or dud (unsuccessful) males. Using these grandchildren, we then measure the following major fitness components: 1) male mating success; 2) a composite measure of adult fecundity and offspring survival to emergence as adults; and 3) adult longevity (because the laboratory stock population is maintained with overlapping generations).

We measure these fitness components on grandchildren of random females mated to stud vs. dud males because indirect genetic benefits, deriving from the superior genes that preferred males may pass on, may arise in generations subsequent to a female's immediate offspring. This may be particularly true for male mating success (attractiveness) because there is some evidence that its genetic basis may involve a disproportionate contribution of the female sex chromosome (i.e. X or Z chromosome). Although the data are mixed and heavily dependent on only two taxa (*Drosophila *and Lepidoptera), the genetic basis of sexual display traits and behavioural reproductive isolation appear to map disproportionately to the X (or Z) chromosome [57-60] (reviewed in [61]). This is consistent with the long hypothesized role of the X chromosome in the evolution of sexual dimorphism [62,63]. A disproportionate contribution of the X chromosome to male attractiveness is of concern because male offspring receive their X chromosome from their mother, not their father. Male grandchildren, however, can trace the origin of their X chromosome to their maternal grandfather 50% of the time on average, and any increased attractiveness resulting from this X will be first manifest in this generation. We therefore use grandchildren of stud and dud males to provide a more powerful test for the existence of any good genes indirect benefit: that is, whether males that are successful at obtaining mates possess superior genes, on average, than do unsuccessful males.

## Results

### Male mating success

**Figure 1:**
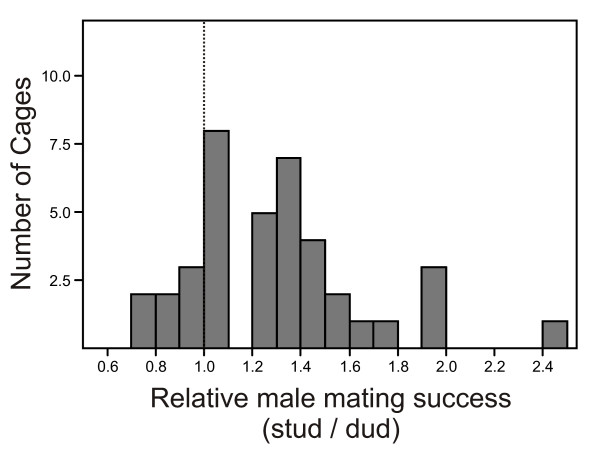
**Frequency distribution of the relative mating success of males from the stud vs dud treatments**. Relative mating successes (proportion of mated males from the stud treatment/proportion of mated males from the dud treatment) were calculated separately for each of the 39 replicate mating cages. The vertical dotted line indicates the expectation under equal mating success. The mating success of males from the stud treatment is significantly greater than that of males from the dud treatment (*p *< 0.0001; see text).

The grandsons of stud males were, on average, 30% more successful at securing mates than were the grandsons of dud males (mean relative mating success of studs vs. duds ± SE: 1.30 ± 0.06; Fig. 1). Treating cages as replicates, this difference in mating success is highly significant (*t*_38 _= 5.16, *p *< 0.0001).

### Productivity

The average productivity (± SE) of individual male-female pairs was slightly greater for the grandchildren of dud males as compared to the grandchildren of stud males when measured at three days (studs: 82.0 ± 1.1, duds: 82.1 ± 1.1) and 15 days (studs: 75.5 ± 1.5, duds: 78.5 ± 1.6) post-emergence. These treatment differences, however, are not significant (Table 1). Productivity declined significantly with age and there was no evidence of an interaction between age and treatment (Table 1).

**Table 1:** Analysis of variance for the productivity of replicate male-female pairs.

Source of variation	df	SS	F	P
treatment	1	14,088,222	2.16	0.142
age	1	59,503,879	9.12	0.003
interaction	1	10,970,819	1.68	0.195
error	726	4,736,707,541		

Sources of variation include the effects of treatment (stud vs. dud ancestry), individual age (three vs. 15 days post-emergence), and their interaction. Productivity is a composite measure that includes the fecundity and fertility of the parents, and the subsequent viability to emergence of their offspring. Productivity values were squared prior to analysis.

### Longevity

**Figure 2:**
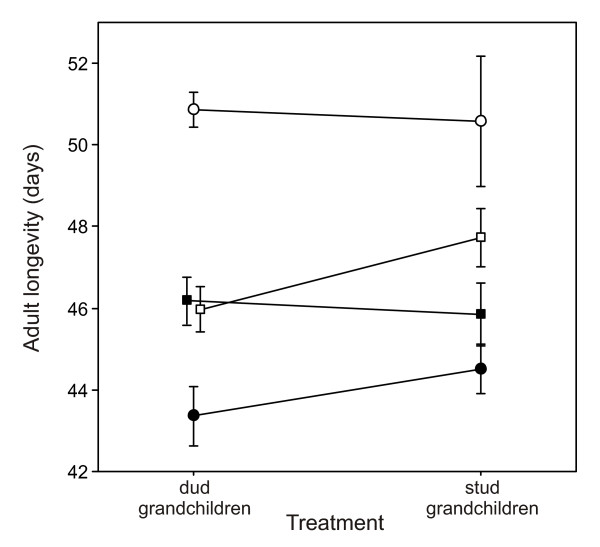
**Mean adult longevity by sex, treatment, and milieu**. Female (circles) and male (squares) longevity was measured in both single sex (open symbols) and mixed sex (closed symbol) milieu. Cages were treated as replicates in all cases. The treatment (stud vs. dud) × milieu interaction is non-significant in both sexes (Table 2). Points are offset in some cases for clarity. Error bars are ± SE.

On average, females lived longer than males when held alone (single sex milieu), but shorter than males when housed under mixed sex conditions (Fig. 2). This effect of milieu in females was highly significant (Table 2). No overall effect of milieu was present in males. In both sexes, treatment (stud vs dud) had no overall effect on mean life span (Table 2), although there was some indication that the grandsons of stud males survived longer than the grandsons of dud males when housed in a single sex milieu (Fig. 2). This treatment × milieu interaction, however, was not significant (p = 0.116; Table 2).

**Table 2:** Results of the general linear models for mean life span (ANOVA) and the two Gompertz mortality parameters (*α*, baseline mortality rate, and *β*, rate of senescence) combined (MANOVA).

	Mean life span				Mortality parameters		
	
Source of variation	df	SS	F	*p*	Wilks' λ	df	*p*
Females							
treatment	1	1.503	0.24	0.625	0.973	2, 26	0.701
milieu	1	356.3	58.01	<0.0001	0.260	2, 26	<0.0001
treatment × milieu	1	3.893	0.63	0.433	0.990	2, 26	0.872
error	27*	529.4					

Males							
treatment	1	4.022	1.18	0.287	0.874	2, 27	0.161
milieu	1	5.406	1.59	0.218	0.700	2, 27	0.0081
treatment × milieu	1	8.977	2.63	0.116	0.684	2, 27	0.0059
error	28	95.41					

*one cage of females (stud, single sex) was lost due to experimental error.

Sources of variation include treatment (stud vs. dud ancestry), milieu (single vs. mixed sexes), and their interaction.

**Figure 3:**
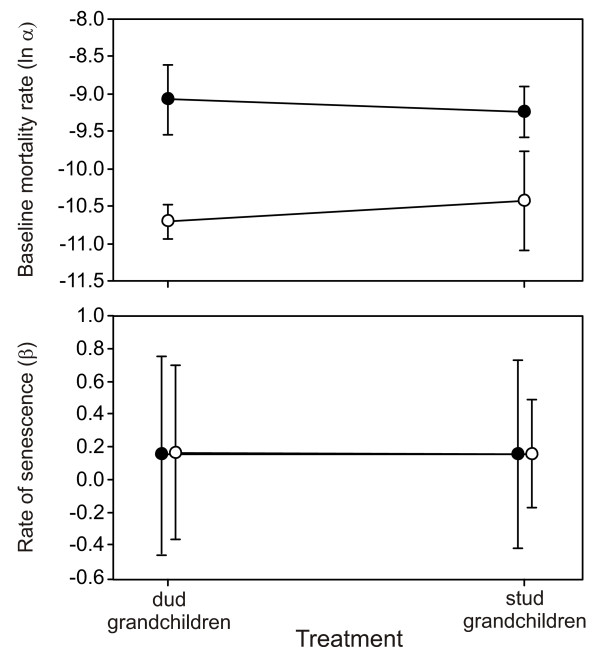
**Mean baseline mortality rate (A) and rate of senescence (B) of replicate cages of females by treatment and milieu**. Milieu are single sex (open circles) and mixed sex (closed circles). Error bars are ± SE.

In line with the results for mean life span, Gompertz mortality parameters for females did not differ significantly between stud and dud treatments, but did differ between single and mixed sex milieu (Fig. 3; Tables 2 and 3). There was no evidence of any treatment × milieu interaction (Table 2). The effect of milieu was caused by an increase in baseline mortality of females in the presence of males (Fig. 3A; ANOVA on Gompertz parameter *α*: F_1,27 _= 10.3, *p *= 0.0035), whereas the presence of males did not appear to affect the rate of senescence in females (Fig. 3B; ANOVA on Gompertz parameter *β*: F_1,27 _= 0.79, *p *= 0.381).

**Table 3:** Summary mortality parameters for males and females for each of N replicate cages.

Sex	Milieu	N (duds/studs)	ln *α *(duds ± SE/studs ± SE)	*β *(duds ± SE/studs ± SE)
females	mixed sex	8/8	-9.08 ± 0.47/-9.25 ± 0.34	0.151 ± 0.010/0.153 ± 0.009
females	single sex	8/7	-10.71 ± 0.22/-10.43 ± 0.66	0.164 ± 0.005/0.156 ± 0.012
males	mixed sex	8/8	-11.14 ± 0.60/-11.28 ± 0.057	0.190 ± 0.013/0.197 ± 0.012
males	single sex	8/8	-10.94 ± 0.53/-9.81 ± 0.33	0.187 ± 0.011/0.153 ± 0.008

Mean (± SE) Gompertz mortality parameters as fit by WinModest [91]. *α *represents baseline mortality and *β *represents the rate of increase of mortality with age (senescence).

**Figure 4:**
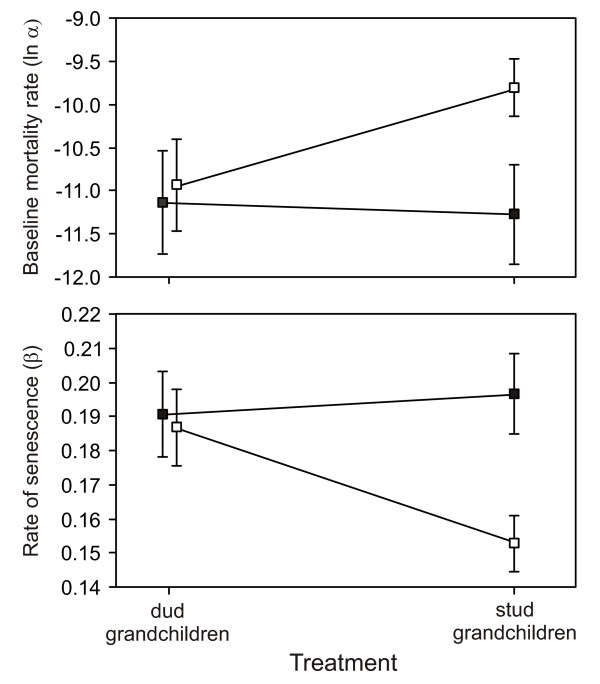
**Mean baseline mortality rate (A) and rate of senescence (B) of replicate cages of males by treatment and milieu**. Milieu are single sex (open circles) and mixed sex (closed circles). Error bars are ± SE.

In males, mortality rates were similar between stud and dud treatments when measured in a mixed sex milieu (their laboratory environment), but when measured in a single sex milieu, males from the stud treatment outperformed males from the dud treatment, having reduced rates of baseline mortality Fig 4A and senescence (Fig. 4B; Table 3). This treatment × milieu interaction was significant overall (Table 2), although it was non-significant when the two mortality parameters were each tested in isolation (ANOVAs testing treatment × milieu interaction for Gompertz parameter *α*: F_1,28 _= 1.48, *p *= 0.235; and *β*: F_1,28 _= 3.27, *p *= 0.081). As noted earlier, this treatment × milieu interaction was also present in average male life span (Fig. 2), although the effect was not significant (Table 2).

## Discussion

Whether females gain any indirect benefit from their choice of mates is a long standing question in evolutionary biology. When present, theory suggests that such benefits may have important implications such as promoting adaptation and increasing population mean fitness [64-68], potentially even providing an advantage to sexual reproduction [69,70]. Empirical evidence for such benefits, however, is mixed, with few experiments addressing both sexual and non-sexual components of fitness in a single design. Here we present the results of an experiment using *D. melanogaster *that evaluated components of both sexual and non-sexual fitness relevant to the laboratory environment to which the population is adapted. Instead of manipulating the opportunity for mate choice with unknown effects on the process of sexual selection, we employed a novel design that allowed us to sample and measure the major components of fitness of individuals with genomes that derived ultimately from males that were either repeatedly successful (studs) or repeatedly unsuccessful (duds) at obtaining mates.

According to our results, individuals with genomes deriving from stud males outperformed individuals with genomes derived from dud males almost exclusively in a single fitness component, male mating success, suggesting that the only indirect benefit to a female in this population of mating with a stud male may arise via an increase in the mating success of her male descendants. This potential sexy son benefit was substantial: males in the stud treatment were 30% more successful on average at securing a mate than males in the dud treatment. In contrast, there was no evidence that productivity (a composite measure of the fecundity and fertility of the parents, and the larval and early adult survivorship of their resulting offspring) differed between individuals from the stud and dud treatments. Similarly for longevity and the mortality parameters determining it, there was no evidence of any differences between stud and dud treatments when measured in a mixed sex milieu (the environment to which the populations are adapted). Surprisingly, however, when held alone (i.e. single sex), males in the stud treatment did have lower rates of baseline mortality and senescence than males in the dud treatment. A benefit of sexual selection in the form of increased longevity has been observed before during experimental evolution in *D. melanogaster *[44], although results were complex with males benefiting independent of milieu whereas females paid a cost when alone but benefited when males were present. In our case, the benefit is small (Fig. 2) and did not translate into a significant effect on longevity (Table 2). It therefore appears unlikely that such a benefit is a key component in the evolution of mate choice in this population.

On one hand, our results are consistent with a previous study using house cricket in which significant indirect benefits were found and that appeared to arise in large part from the increased attractiveness of male offspring [54]. On the other hand, in apparent contrast to our results, a series of experiments using the LH_M _population of *D. melanogaster *failed to find any significant indirect benefits, including sexy sons [17,18,55,56,71], despite previous work demonstrating the heritability of lifetime fitness [16]. (For a review of this work, see [72].) A number of these experiments, however, compared treatments that varied the strength of sexual selection [17,18,56], thereby estimating an absolute cost of sexual selection. Our experiment, in contrast, addressed the relative costs and benefits of mating with different males (stud vs. dud); the presence of an absolute cost was not addressed. Nevertheless, at least two past studies using the LH_M _population examined the fitness effects of male identity [55,71], thereby addressing a relative cost/benefit. The explanation for these contrasting results in the same species is unknown, although it could represent among-population variation in the costs and benefits of mate choice. Alternately, it could trace its origin to difficulties in the empirical measurement of lifetime fitness using fitness components – important components, including male mating success, may been overlooked [73]. We return to this topic later.

What role might these indirect benefits play in the evolution of female mate preferences in our population? Answering this question will require comparable estimates of the direct costs of mating with successful vs. unsuccessful males, as well as information about how these costs and benefits integrate across generations. There are two main possibilities, however. First, these indirect benefits may outweigh any direct costs and mate preferences may therefore represent adaptations in females to gain these benefits. According to theoretical analyses, however, a pure sexy sons process, in which the only benefit to females of mating with attractive males comes from the increased mating success of sons [29,51], cannot maintain a costly female preference at equilibrium [29,30,74]. Although there is evidence from various taxa that mate preferences are costly [75,76], it is possible that they have little or no cost in our *D. melanogaster *laboratory population, where females can readily sample numerous males and rejected unwanted males with little effort. Determining the cost of female preferences is an empirical issue, albeit a difficult one, that therefore demands attention if we wish a comprehensive understanding of the nature of indirect benefits.

Alternatively, indirect benefits to females in this population may have arisen through the production of offspring of superior genetic quality, and hence higher condition (i.e. a good genes process), with males simply allocating their surplus condition primarily to traits that increase their mating success. Mating success may be a dominant component of male fitness in laboratory *Drosophila *populations, although the maintenance of our population with overlapping generations would be expected to make longevity an additional key component. Consistent with such a good genes scenario, when denied the opportunity to mate due to the complete absence of females, males from the stud treatment lived longer than males from the dud treatment, consistent with the idea that they may have shifted their allocation of surplus condition to traits affecting longevity in that particular environment. However, such a good genes scenario also predicts that females from the stud treatment should have been of superior genetic quality, and thus have had higher condition on average, when compared with females from the dud treatment. The lack of any detectable difference in female fitness between our stud and dud treatments suggests that this is not the case.

The second possibility is that these indirect benefits do not outweigh the direct costs of mating in this population and mate choice is therefore dominated by sexual conflict. Under such a scenario, instead of indicating attractiveness, variation in male mating success (i.e. stud vs dud males) may reflect differences in the ability of males to coerce or otherwise cause females to mate beyond their natural selection optimum, and the ability of females to resist this. Females need not benefit indirectly in any way from their 'choice' of males and 'mate preferences' in females, which may be more accurately construed as varying levels of resistance, may evolve because they minimize direct costs of mating arising from this sexual conflict [4]. There is ample evidence that sexual conflict dominates in the LH_M _population of *D. melanogaster *[9-11,16-18]. However, in our population, being housed with males from the stud vs. dud treatments had no differential effect on the longevity or mortality rates of females, nor did female productivity differ when mated with either type of male. Nevertheless, we do not know what effect, if any, mating with these two types of males may have on the longer term reproductive success of females (i.e. productivity from future matings), and we lack data on the absolute cost of sexual selection. Determining whether such absolute costs exist will be key to understanding the role of sexual conflict in the evolution of mate choice in this population.

As the above discussion highlights, the evolution of mate choice depends ultimately on the net fitness effects of mating with 'preferred' males, including both direct effects on the individuals and indirect effects arising from variation in the sexual and non-sexual fitness of their descendants. Simultaneous measurement of direct and indirect fitness effects is an empirical challenge that has rarely been attempted. As recently demonstrated in *D. melanogaster *[18], experimental evolution, in which the organisms themselves integrate the relevant fitness components over multiple generations in their representative environment, may be a powerful approach for achieving this goal [4]. In this case, the evidence supports the latter scenario of sexual conflict. (The extent to which, if any, sexual conflict is enhanced in laboratory populations as compared to populations from nature becomes an important question.) Valuable insight may also be gained from long-term studies of populations in nature [45,77,78] in which an extensive pedigree is known, long-term reproductive success can be measured in the wild, and quantitative genetic parameters can be estimated. In addition to such 'net fitness' approaches, however, a comprehensive understanding of the evolution of mate choice will require an understanding of how individuals allocate condition to various fitness components and how this varies with environment. Detailed experimental estimates of the various direct and indirect costs and benefits that integrate to determine net fitness will therefore remain a difficult yet important endeavour in sexual selection research.

## Conclusion

Individuals with genomes deriving ultimately from males that were successful at obtaining mates tended to outperform individuals with genomes deriving ultimately from males that were unsuccessful at obtaining mates, suggesting the existence of an indirect benefit of mating with successful males in this population. This advantage, however, came almost exclusively in the form of a single fitness component: male mating success. It is important to note, however, that as with all studies that measure components of fitness, it is possible that individuals may allocate condition differently under different situations; the absence of indirect longevity or productivity benefits in our experiment cannot therefore preclude their absence in this population in other environments. Therefore, whether our results represent a traditional sexy sons benefit or are also indicative of a good genes process in which surplus condition was being allocated by males primarily to enhance their mating success, is a crucial question that remains. Future studies are needed that address how allocation to various life history components varies with environment. It will also be important to estimate the direct costs of mating in this population, and to determine how the various costs and benefits integrate across generations. Although experimentally challenging, such work promises to shed light on the selective mechanisms responsible for the evolution of female 'mate preferences'.

## Methods

### Stock population

A stock population of *Drosophila melanogaster *was initiated in June 2002 from a large (~100 females) sample of flies collected from Commercial Avenue in downtown Vancouver, BC, Canada. Since its collection, this stock has been kept in a minimum of four population cages (37 × 27 × 21 cm; L × W × H) at a large census size (> 5000 flies) under constant conditions (50% relative humidity, 12L:12D photoperiod, 25°C) with overlapping generations. As a source of food and water, two standard 240 ml bottles, each containing 50 ml of sucrose-cornmeal media with live yeast sprinkled on top, are placed in each cage and replaced weekly. New adults are produced every week by allowing approximately 300 eggs to be laid in a new bottle added to each cage. These bottles are then removed for one week and then returned to a cage for one additional week. This procedure truncates the development time of individual flies to14 days. This stock has been used in several investigations [79-82] and shows vigorous behaviour in the lab in comparison with other *D. melanogaster *stocks (Dukas and Mooers, pers. obs.).

### Generating stud/dud treatment males

**Figure 5:**
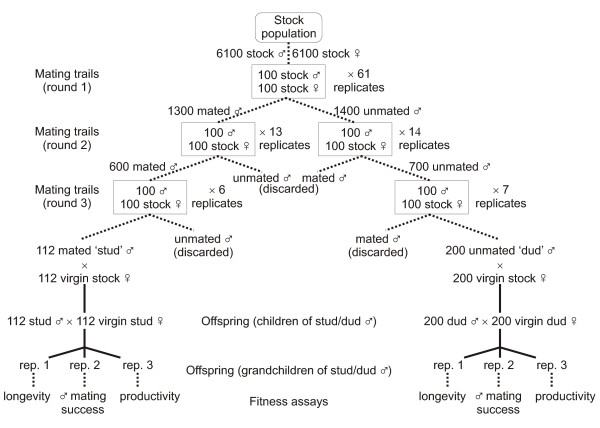
**Experimental protocol used to sort males into studs and duds and then produce grandsons**. Broken lines connect experimental procedures within a generation and solid lines connect parental and offspring generations.

The experiment was initiated on September 30, 2003. The protocol used non-virgin 'generator females', taken from the stock population, in three consecutive rounds of mating trails to sort stock males into two groups: 'studs', those which were successful at obtaining a mating during each of the three rounds, and 'duds', those which were unsuccessful at securing a mating during each of the three rounds (Fig. 5). (Non-virgin females were used because theory [83,84] and empirical data [85,86] both suggest the possibility that, in general, virgin females of various taxa may be less discriminating in their choice of mates.) Generator females were produced by allowing stock flies to eclose into cages (10 days after they were laid as eggs) and then to mate freely until the morning three days hence. At this time, flies were separated by sex using light CO_2 _anaesthesia and females were held in bottles of 50 flies with 50 ml media and live yeast sprinkled on top. When these females were 8–9 days old (5–6 days after their last possible mating), they were used in mating trials and then discarded. The creation of generator females from the stock population was done repeatedly in a staggered manner such that new 8–9 day-old non-virgin adult females (held 5–6 days without mates) were available for each of the six days that mating trials were performed. The males that were sorted into studs and duds during the three rounds of mating trails were originally collected from the stock population together with the generator females used in the first round. Prior to the mating trails, these males were stored in the same manner as the females and during the first round of mating trails were the same age (8–9 days post-emergence) as the females and had been held for 5–6 in the absence of females.

Mating trials were conduced in Plexiglas population cages (37 × 27 × 21 cm^3^, covered on all exterior sides except the top with white paper (to minimize disturbance of the flies) at ambient temperature and humidity (22–24°C, 35–55% relative humidity) between 0900–1200 h. Each trail involved adding approximately 100 males and 100 females to a single cage and then collecting, by aspiration, all pairs found copulating (stud males) during two visual inspections (at 12 and 24 minutes respectively), and subsequently, all males that did not commence mating within 24 minutes (dud males). Immediately following the trials, stud males from the two collections were pooled and separated from their mates (generator females) using light CO_2 _anaesthesia and then stored in bottles of 50 until the next round of mating trials 2–3 days later (females were discarded). Dud males were given a similar dose of anaesthesia to match that received by the stud males and then stored as above. In rounds two and three of the mating trials, previously successful males that failed to mate, and previously unsuccessful males that obtained a mating, were discarded such that only repeatedly successful (stud) or repeatedly unsuccessful (dud) males were retained.

The first round of mating trails took place over three days and generated 1300 stud males and 1400 dud males overall from 61 replicate mating cages. These males were held for two additional days after the end of this round, after which time they were used in the second round of mating trials conducted over two days (13 replicate cages performed using only round-one stud males and 14 using only round-one dud males). From this second round of trials, 600 round-two stud males (males successful in rounds one and two) and 700 round-two dud males (males unsuccessful in rounds one and two) were collected and held as before. After three days, these males were used in the third and final round of mating trials, conducted in a single day. From this third round, 112 stud males (three-times successful) and 200 dud males (three-times unsuccessful) were obtained from six and seven replicate mating trials respectively. At this point, the males were 15–16 days post-emergence. In addition to designating males as either studs or duds, our sorting technique also selected for males that survived to 15–16 days post-emergence and that were reproductively competent (see below). However, this sorting was constant between stud and dud males (i.e. in both treatments, only males that survived to 15–16 days post-emergence and were reproductively competent contributed to the next generation – see below).

Offspring from these males were then generated by mating each male to a random, virgin female collected from the stock population and then allowing each female to lay approximately 50 eggs in a fresh vial over a 24 h period. Once these offspring had emerged, random pairs of stud (dud) adults were created by taking a virgin male from one stud (dud) vial and a virgin female from another stud (dud) vial and placing them together in a new vial for mating. After 24 h, these pairs were transferred to new vials for egg laying. Subsequent transfers were performed after 24 h each to produce three replicate sets of stud and dud grandchildren (replicates 1–3; Fig. 5) using adults that were 11, 12, and 13 days post-emergence. Fitness components of these grandchildren of stud and dud males were then measured as described below, using one of the three replicate sets of stud and dud grandchildren for each of the three fitness components considered (replicate number was the same between the stud and dud treatments for each fitness measure).

### Measurement and analysis of fitness components of grandchildren

#### Male mating success

2250 male and 2250 female grandchildren were collected from replicate 2 studs and duds respectively and allowed to mate among themselves in population cages for two days before males were collected (females were discarded) using light CO_2 _anaesthesia and stored in groups of 50 flies/bottle on food with no live yeast added. Mating trails were conducted six days later (eight days post-emergence, i.e. at the age their grandfathers were first tested). Twelve hours prior to the trials, stud and dud males were placed in vials containing abundant live yeast impregnated with red or blue food colouring ("Food-club Brand", Scott-Bathgate Ltd) in a near balanced design (18 of the subsequent mating cages had red studs/blue duds and 21 had the reverse). The coloured yeast that the males eat is easily visible through their abdomen, thereby marking them temporarily either red or blue. Extensive use of such marking in previous experiments has shown no effect on mating patterns [87-89], nor was there any evidence of non-random mating by colour in the current experiment; of the 1,712 observed matings, 48.7% involved red males, which does not differ from random expectation (χ^2^_c_-test with p^
 MathType@MTEF@5@5@+=feaafiart1ev1aaatCvAUfKttLearuWrP9MDH5MBPbIqV92AaeXatLxBI9gBaebbnrfifHhDYfgasaacH8akY=wiFfYdH8Gipec8Eeeu0xXdbba9frFj0=OqFfea0dXdd9vqai=hGuQ8kuc9pgc9s8qqaq=dirpe0xb9q8qiLsFr0=vr0=vr0dc8meaabaqaciaacaGaaeqabaqabeGadaaakeaacuWGWbaCgaqcaaaa@2E25@ = 0.5, P = 0.27).

Females for use in the mating trials were created by collecting males and females from the stock population and allowing them to mate in population cages. After two days, the stock females were separated by sex using light CO_2 _anaesthesia and stored separately for five days. Thirty-nine replicate mating trials (cages) were performed on a single day between 0900–1330 h. In each trial, 50 marked stud males and 50 alternately marked dud males were placed together with 100 stock females in a cage. Mating pairs were removed by aspiration as before using two visual observations at 12 and 24 minutes and the males were then identified by colour. In mating trials such as these, separate matings within a cage are not independent of one another because the relative frequency of the different types of flies change as the trial proceeds and individuals mate. We therefore treated individual cages as replicates and, for each cage, calculated the relative mating success of stud vs. dud males by dividing the proportion of successful studs (# mated studs/total # of studs) by the proportion of successful duds (# mated duds/total # of duds). This controlled for slight variation in the total numbers of stud and dud males in a cage (because of the odd fly dying or escaping). After natural-log transformation, this measure was normally distributed so a *t*-test (treating cages as replicates) was used to compare the sample mean to *ln*(1), the expected value under the null hypothesis of equal mating success of the male grandchildren of stud and dud males. Results are qualitatively similar if raw counts are used in place of ratios in the analysis.

#### Productivity & Offspring Viability

700 male and 700 female virgin grandchildren from both stud and dud males were collected from replicate 3 vials and stored separately by sex in bottles of 50 flies with yeasted food (changed weekly). The productivity of these grandchildren was measured at two ages (three and 15 days post-emergence), as follows. Forty-eight hours prior to measuring their productivity, flies were taken from their storage bottles and placed as single male-female pairs in vials for mating. After 48 h in these vials, pairs were transferred simultaneously by tandem workers to new yeasted vials for exactly 24 h of egg laying, after which they were removed and discarded in parallel for the two treatments (studs and duds). Using this protocol, 193 (184) replicate stud and 194 (189) replicate dud vials were created from the eggs produced by 3 (15) day-old male-female pairs of stud and dud grandchildren. In each case, 11 days after their creation, these productivity vials were frozen and the number of adults (great-grandchildren of stud and dud males) that had emerged was subsequently counted separately for each vial.

Productivity, measured as the number of adult offspring produced, represents a large component of total fitness because it is a composite measure of the fecundity and fertility of the parents, and the larval and early adult survivorship of their resulting offspring. This measure, however, was bimodally distributed because some stud and dud pairs at both ages failed to produce offspring. Because this fraction was small (3.4% overall from the studs and 4.4% from the duds) and we did not know why these pairs failed to produce offspring (possibilities include an infertile male and/or female, refusal to mate, and experimenter error), analyses were restricted to those pairs that produced offspring. Results do not change qualitatively when nonparametric analyses are applied to the total (non-normal) data, including zeros. Non-zero productivity data were transformed by squaring prior to analysis to normalize their distributions. Differences in productivity were tested using a general linear model (ANOVA) with the productivity count of individual vials as replicates and treatment (stud vs. dud), parent's age (3 vs. 15 days old), and their interaction as fixed effects. Dropping the non-significant interaction had little effect on the significance of either main effect.

#### Longevity

Grandchildren from replicate1 vials were allowed to emerge into cages (10 days after being laid as eggs). These flies were then sexed using light CO_2 _anaesthesia and a pair of workers set up eight replicate 750 ml clear plastic longevity cages [cages are described in [90]] for each of three treatment milieus (75 pairs/cage, 75 males/cage, and 75 females/cage) simultaneously for both stud and dud treatments, yielding a total of 48 cages. These cages were placed in alternate order in rows in the same incubator in which the stock flies were kept. Every 48 hours thereafter (excepting a single 72 hour interval), food vials were changed for each cage and dead flies were removed and identified by sex until there were no live flies remaining.

After censoring the first mortality count to remove any incidental effects associated with the transfer of flies to the longevity cages, the remaining longevity data were analysed separately by sex, treating individual cages as replicates. Differences in mean life span were tested using a general linear model (ANOVA) with treatment (stud vs. dud), milieu (single vs. mixed sex), and their interaction as fixed effects. Because differences between treatments in the temporal patterns of mortality can exist in the absence of mean life span differences (e.g., a treatment could increase initial mortality but lower the rate of senescence, having no net effect of mean longevity), we also tested mortality parameters directly. The best-fit mortality model from the Gompertz family was first determined using the maximum likelihood method implemented in the software package WinModest [91]. In the majority of cages, mortality was best described by the Gompertz model [92]: *μ*_*x *_= *αe*^*βx*^, where *μ*_*x *_is the predicted instantaneous mortality rate at age *x*, *α *is the baseline mortality rate, and *β *is the rate of senescence (i.e. the rate at which mortality increases with age). We therefore used WinModest to estimate *α *and *β *separately for each cage using the Gompertz model. Baseline mortality rate values were *ln*-transformed prior to analysis to normalize their distributions. Differences in mortality parameters (*α *and *β*) were tested using a multivariate general linear model (MANOVA) with treatment (stud vs. dud), milieu (single vs. mixed sex), and their interaction as fixed effects. When multivariate significance was present, separate analyses of variance were used to explore the effects on each of the two mortality parameters.

## Authors' contributions

HDR and AØM conceived of the study. All authors helped design and conduct the experiment and perform the statistical analyses. HDR drafted the manuscript. All authors read and approved the final manuscript.
